# Nonsurgical treatment of an adult with skeletal Class III malocclusion, posterior crossbite and mandibular asymmetry

**DOI:** 10.4317/jced.60939

**Published:** 2023-11-01

**Authors:** Carlos Bellot-Arcís, Elena Ferrando-Magraner, Beatriz Tarazona-Álvarez, Verónica García-Sanz, Vanessa Paredes-Gallardo

**Affiliations:** 1 Associate Professor, Department of Stomatology, Faculty of Medicine and Dentistry, University of Valencia, Spain; 2 PhD Candidate; Orthodontist. Department of Stomatology, Faculty of Medicine and Dentistry, University of Valencia, Spain; 3 Assistant Professor. Department of Stomatology, Faculty of Medicine and Dentistry, University of Valencia, Spain; 4 Research Associate and Lecturer. Department of Stomatology, Faculty of Medicine and Dentistry, University of Valencia, Spain; 5 Associate Professor, Department of Stomatology, Faculty of Medicine and Dentistry, University of Valencia, Spain

## Abstract

Class III malocclusion represents a very heterogeneous clinical condition that is characterized by the combination of a wide variety of skeletal and/or dental components. Given the wide diversity, diagnosis and treatment of such malocclusion has always been a challenge for clinicians. Despite the different treatment options available, the treatment approach in the adult patient must depend fundamentally on the patient’s decision, guided by the orthodontist and the maxillofacial surgeon. This case report presents the treatment of a patient with Class III malocclusion, with posterior crossbite and anterior edge-to-edge bite with fixed appliances and skeletal anchorage, an interdisciplinary, nonsurgical approach for a skeletal malocclusion. Firstly, to improve the posterior transverse relationship a band-soldered compressed lingual arch was cemented to the mandibular first molars. Then, once a correct transverse relationship was achieved, two miniscrews were placed distal to the mandibular second molars to distalize the whole mandibular arch, and avoid excessive inclination of maxillary incisors to improve dentofacial esthetics. At the end of the treatment, all the objectives planned at the beginning had been achieved and remained stable after the retention period.

** Key words:**Clase III, orthodontic treatment, distalizing, appliances, posterior crossbite.

## Introduction

Class III malocclusion is a very heterogeneous clinical condition characterized by the combination of a wide variety of skeletal and/or dental components (1-3). Given the wide diversity of malocclusions, diagnosis, prediction and treatment, of such malocclusion has always been a challenge for clinicians. Achieving correct occlusion and improving facial aesthetics are the main objectives in the treatment of class III, which can be achieved through different treatment options; growth modification, orthodontic camouflage, or orthognathic surgery combined with orthodontic treatment (2,4,5).

The non-surgical treatment of adult patients with a Class III malocclusion has traditionally been planned with fixed orthodontic appliances combined with intermaxillary elastics to achieve distal movement of the mandibular molars. This approach can be unpredictable, since it requires the collaboration of the patient, as well as generating certain instability and aesthetic alteration, as a consequence of the appearance of undesirable effects generated by the use of elastics (6). However, the appearance of skeletal anchorage has made it possible to improve the approach and results of non-surgical treatment of skeletal class III, and even to achieve an aesthetic benefit for these patients. Miniplates and miniscrews provide sufficient anchorage to generate orthodontic dental movement, avoiding unwanted secondary dental effects derived from the use of intermaxillary elastics (6,7).

Maxillary involvement in most Class III cases manifests with maxillary hypoplasia, not only in the sagittal plane but also in the transverse dimension, which is accompanied by the appearance of both anterior and posterior crossbite in many cases (8).

This case report detail the orthodontic treatment of a patient with a skeletal Class III, posterior crossbite in the right side and anterior edge-to-edge bite, treated with fixed bonded appliances and skeletal anchorage to retract the whole mandibular dentition, avoiding excessive protrusion of the maxillary incisors, and thus improve facial aesthetics.

## Case Report

-Diagnosis and etiology

A 39-year-old female patient attended the consultation after being referred for orthodontic evaluation, referring the following reason (in her own words): “I think I don’t bite correctly; I don’t close the same on both sides”.

**Figure 1:**
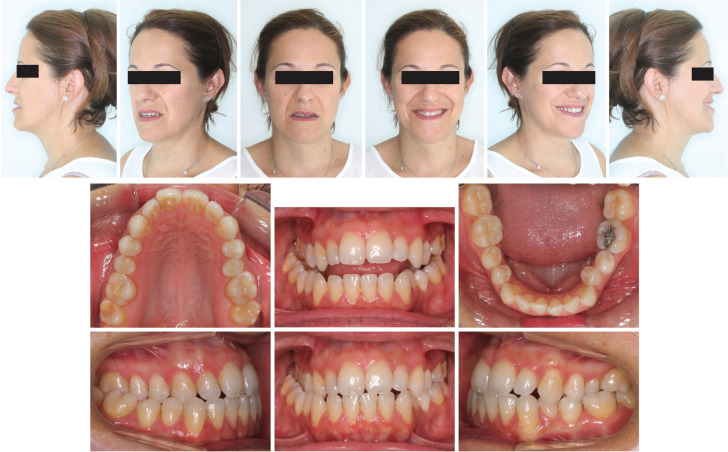
Pre-treatment facial and intraoral photographs.

Extraorally, the patient presented a mesofacial pattern with a proportionate but asymmetrical face, the nose and chin deviated to the right. A straight profile presented with a tendency to concave, in which a slightly everted lower lip stood out. When analyzing the patient’s smile, maxillary compression stood out. The exposure of the incisors was slightly diminished at rest, partially exposing the lower incisors (Fig. 1).

Intraorally, the patient presented a right Class I molar and canine relationship and a left Class III molar and canine relationship, with maxillary incisors slightly proclined. Transversely, she presented a right posterior crossbite that compromised the entire posterior sector; however, on the left side only presented a dental crossbite at the level of the upper second premolar. The patient presented anterior edge-to-edge relationship, with decreased overjet (1 mm) and decreased overbite (0 mm). The maxillary dental midline was centered with the face, with the mandibular midline deviated 4mm to the right. The maxillary arch presented a triangular morphology with the right side compressed, in correlation with the posterior crossbite; the maxillary arch showed a more quadrangular tendency (Fig. 1).

The cephalometric analysis (Table 1) allowed us to identify a skeletal Class III (ANB – 1.8º; Wits – 5.2 mm) and a mesofacial growth pattern (facial axis 92.1º; FMA 29.2º). In relation to the inclination of the incisors, in the maxillary arch they were proclined (U1-palatal plane 114º), and in the mandibular arch they were retroclined (IMPA 82.2º).

**Table 1:**
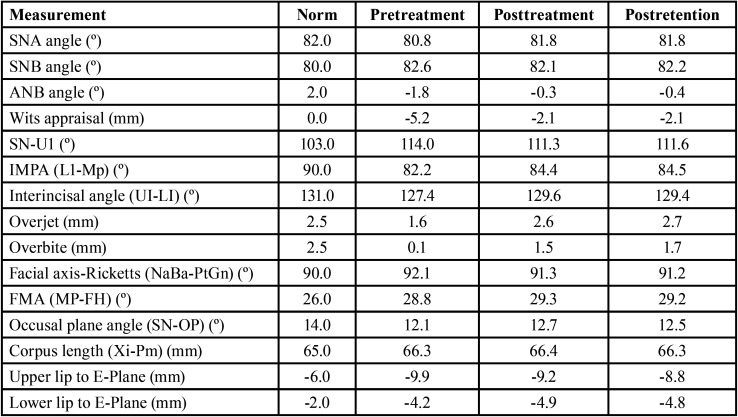
Cephalometric values.

The functional analysis did not showed a variation in terms of centric relation, the posterior crossbite and the anterior edge-to-edge contact did not change in centric relation, a reflection of the skeletal mandibular asymmetry that the patient presented, as previously described.

-Treatment objectives

The planned treatment objectives were aimed at: improving the transversal relationship between both jaws through mandibular dental compression and maxillary dental expansion, achieving a Class I dental relationship through distalization of the mandibular arch using skeletal anchorage, obtaining an adequate overjet and overbite, as well as establishing correct torque of maxillary and mandibular incisors, and correct facial aesthetics.

-Treatment progress

The first part of the treatment was based on improving the posterior transverse relationship; 0.022 x 0.028-inch Tip-Edge Plus brackets (TP Orthodontics Inc) were cemented throughout the maxillary arch, from second molar to second molar, and work began with nickel-titanium (NiTi) archwires to favor the alignment, leveling and transversal development of the maxillary arch. In the mandibular arch, a band-soldered compressed lingual arch (0.36-inch wire) was constructed and cemented on the mandibular first molars. The mandibular fixed appliances made it possible to compress the mandibular first molars and generate a crown-lingual inclination of said pieces, and later compress the entire mandibular arch taking the first molars as anchorage.

**Figure 2:**
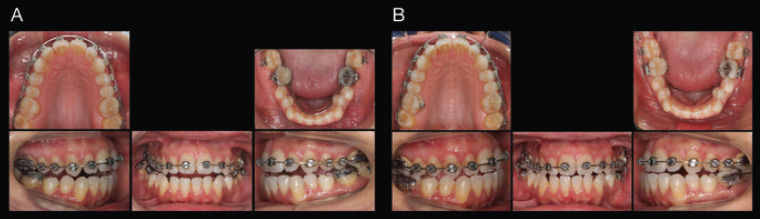
Mechanics used to develop the maxillary arch and to compress the mandibular arch; Niti wire 0.017 x 0.025 inches in the maxillary arch and compressed lingual arch in the mandibular arch (A). Mechanics used to compress and align the mandibular second molars; Niti arch 0.016 x 0.022. and miniscrews placed distal to the second molars (B).

Four months after the placement of the lingual arch, a correct transverse relationship of the first mandibular molars had been achieved. At this time, fixed appliances were cemented on of the second mandibular molars and, taking the first molars as anchorage, using sectional wires of a nickel-titanium alloy (Fig. 2A). The mandibular second molars were compressed and aligned with the first molars.

Subsequently, once a correct transverse relationship was achieved at the molar level, two miniscrews were positioned in the retromolar area, distal to the mandibular second molars (length, 12 mm; diameter, 1.2 mm; AbsoAnchor [Dentos, Daegu, South Korea]) (Fig. 2B). The miniscrews functioned as a direct anchor that allowed distalization of the whole mandibular arch, the distal traction force was applied with elastic chains anchored to the miniscrews, only in the buccal location to help compression of the mandibular arch (9).

Four months after starting the distalization of the mandibular arch spaces began to appear mesial to the molars and the sagittal position of the molars had improved. At that time, the fixed mandibular arch appliances were cemented. For the alignment and leveling of the arches, 0.016-in, 0.016 x 0.025-in and 0.021 x 0.025-in NiTi wires were used, as well as 0.019 x 0.025-in and 0.021 x 0.028-in stainless steel wires to finish the flattening and compensate the anterior dental torque.

The treatment lasted for 26 months. After removal of the orthodontic devices, a fixed retainer was placed on the lingual surface of the anterior teeth and a removable thermoplastic retainer was prepared and a nocturnal use of the same was indicated. Consent was obtained from the patient for the publication of the case progress records.

## Results

**Figure 3:**
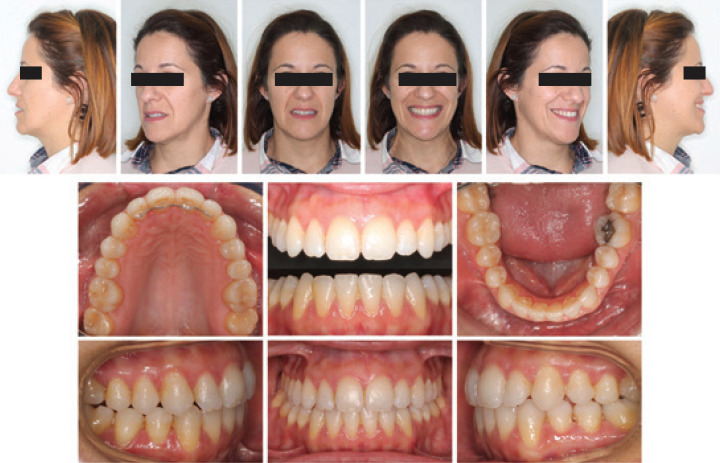
Post-treatment facial and intraoral photographs.

The evaluation of the final records of the patient reflects that all the planned treatment objectives were achieved, resulting in a correct occlusion and an improvement in facial aesthetics. Aligned, leveled, and coordinated arches were achieved, which allowed the resolution of the right crossbite, achieving a correct transversal relationship between both arches (Fig. 3).

A complete correction of the sagittal problem was achieved through the use of skeletal anchorage, it was not necessary to use Class III elastics, which made it possible to avoid possible adverse effects derived from inter-arch anchorage, such as proclination of the maxillary incisors.

Intraoral photographs and end-of-treatment casts showed that a Class I molar and canine relationship had been achieved, with normalized overbite and overbite (Fig. 3). The facial photographs after the treatment showed a proportionate facial aesthetics, with a harmonious profile and a wide and consonant smile (Fig. 3).

The cephalometric analysis allowed to identify significant changes. The ANB value dropped from -1.8º to -0.3º and the Wits value ranged from -5.2 mm to -2.1 mm. Despite the inclination of the incisors improving, the maxillary incisors remained slightly proclined (SN-U1 from 114.0º to 111.3º) and the mandibular incisors slightly retroclined (IMPA from 82.2º to 84.4º) (Table 1).

**Figure 4:**
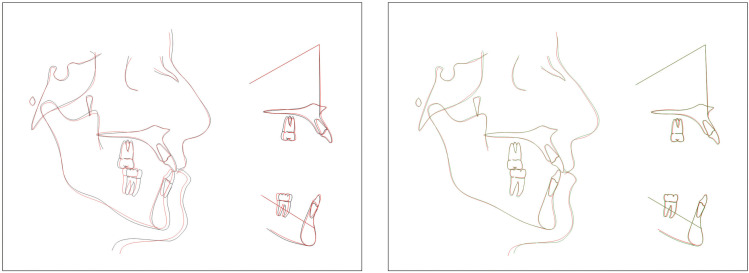
Superposition of the pre-treatment (black line) and post-treatment (red line) cephalometric tracings (A); Overlay of post-treatment (red line) and post-retention (green line) cephalometric tracings (B). Superimposed in the sella-nasion plane in the sella, in the palatal plane and in the mandibular plane.

The superimposition of the initial and final records shown in Figure 4 illustrates that the correction of the malocclusion was achieved due to the distalization and intrusion of the mandibular dentition. Similarly, it reflects that there was no proclination of the maxillary incisors.

12 months after the end of the treatment, the occlusion achieved remained unchanged at the transverse, sagittal, or vertical level.

## Discussion

The diversity of involvement raises a wide range of treatment possibilities ranging from surgical treatments to growth modification (4,10). However, clinicians may often overlook a fundamental factor when considering the most appropriate treatment option, and on which the treatment decision will ultimately depend: the opinion of the patient. The dilemma we face in many cases of severe malocclusions is that most of these patients reject or are not willing to accept surgical therapy and insist on orthodontic treatment, despite being the improvement in facial aesthetics the main reason for consultation (11).

In the present case, despite presenting a skeletal class III malocclusion with an anterior edge-to-edge bite and a posterior crossbite, the patient did not present a significant aesthetic compromise, and the improvement of facial aesthetics was not one of the reasons for consultation. That is why, after the examination and diagnosis of the patient, camouflage orthodontic therapy was chosen.

The main treatment objectives were to improve the transverse and sagittal relationship between both arches, without compromising the patient’s facial aesthetics. Regarding the sagittal relationship, a fundamental aspect to consider during treatment planning is the labiolingual inclination of the maxillary incisor; normally, patients with class III present at the time of diagnosis proclined maxillary incisors, which should not be projected any further, since excessive proclination of the maxillary incisors as a mechanism to compensate maxillary hypoplasia is unsightly and worsens the patient’s facial profile (11-13). In such cases, the treatment plan should focus on mandibular arch retraction, either through skeletal anchorage, or through extractions in severe cases, but always considering the skeletal discrepancy and the labiolingual position of the mandibular incisors (13). In the present case, a therapeutic option using skeletal anchorage was chosen, since despite presenting a severe class III dental relationship in the left hemiarch with a significant deviation from the lower midline, the osseous-dental discrepancy of the mandibular arch did not it was very sharp. In addition, it was a more conservative approach, the patient did not present the wisdom teeth, they had been previously removed for non-orthodontic purposes, so the extraction of other teeth would have meant leaving the maxillary second molars without occlusion.

The distalization of the mandibular arch by means of miniscrews allowed to achieve a correct sagittal relationship, adequate inclination of the incisors and a satisfactory aesthetics. The use of class III intermaxillary elastics was not necessary at any time, avoiding the adverse effects derived from the use of elastics to achieve sagittal compensation (excessive proclination and relative intrusion of the maxillary incisors, or retroclination of the mandibular incisors) (5,14). In the same way, not considering the option of mandibular to compensate the skeletal class III, the excessive retroclination of the mandibular incisors and the impact that it entails at the level of smile aesthetics were avoided (15).

At the transverse level, and as a consequence of the maxillary hypoplasia, the patient presented a right posterior crossbite that was completely corrected by using auxiliary fixed appliances (compressed lingual arch) and distalization of the mandibular arch. A dental-anchored device was chosen, knowing and assuming the possible consequences that this could entail (limited skeletal expansion, crown-lingual inclination undesirable damage to the teeth, possible buccal bone dehiscence) (14). However, the combination of the auxiliary fixed appliances together with the distalization of the mandibular dentition, the dento-alveolar expansion of the maxillary arch achieved through the fixed appliances and the use of cross-bite elastics, allowed the maintenance of correct bone support and periodontal considerations, as well as achieving a proportionate vestibular-palatal inclination of both the maxillary and mandibular posterior teeth.

Post-retention records demonstrate that fixed orthodontic appliance treatment combined with skeletal anchorage and auxiliary fixed appliances remained stable after 1 year of retention.
